# Identification of loci associated with pathological outcomes in Holstein cattle infected with *Mycobacterium avium* subsp. *paratuberculosis* using whole-genome sequence data

**DOI:** 10.1038/s41598-021-99672-4

**Published:** 2021-10-11

**Authors:** Maria Canive, Gerard Badia-Bringué, Patricia Vázquez, Oscar González-Recio, Almudena Fernández, Joseba M. Garrido, Ramón A. Juste, Marta Alonso-Hearn

**Affiliations:** 1 Department of Animal Health, NEIKER-Basque Institute for Agricultural Research and Development, Basque Research and Technology Alliance (BRTA), Derio, Bizkaia Spain; 2 Doctoral Program in Immunology, Microbiology and Parasitology, Universidad del País Vasco/Euskal Herriko Unibertsitatea (UPV/EHU), Leioa, Bizkaia Spain; 3 Doctoral Program in Molecular Biology and Biomedicine, Universidad del País Vasco/Euskal Herriko Unibertsitatea (UPV/EHU), Leioa, Bizkaia Spain; 4 Departamento de Mejora Genética Animal, Instituto Nacional de Investigación y Tecnología Agraria y Alimentaria, CSIC, Madrid, Spain; 5 Departamento de Producción Agraria, Escuela Técnica Superior de Ingeniería Agronómica, Alimentaria y de Biosistemas, Universidad Politécnica de Madrid, Ciudad Universitaria, Madrid, Spain

**Keywords:** Bacterial host response, Genome-wide association studies

## Abstract

Bovine paratuberculosis (PTB), caused by *Mycobacterium avium* subsp. *paratuberculosis* (MAP), is a chronic granulomatous enteritis that affects cattle worldwide. According to their severity and extension, PTB-associated histological lesions have been classified into the following groups; focal, multifocal, and diffuse. It is unknown whether these lesions represent sequential stages or divergent outcomes. In the current study, the associations between host genetic and pathology were explored by genotyping 813 Spanish Holstein cows with no visible lesions (N = 373) and with focal (N = 371), multifocal (N = 33), and diffuse (N = 33) lesions in gut tissues and regional lymph nodes. DNA from peripheral blood samples of these animals was genotyped with the bovine EuroG MD Bead Chip, and the corresponding genotypes were imputed to whole-genome sequencing (WGS) data using the 1000 Bull genomes reference population. A genome-wide association study (GWAS) was performed using the WGS data and the presence or absence of each type of histological lesion in a case–control approach. A total of 192 and 92 single nucleotide polymorphisms (SNPs) defining 13 and 9 distinct quantitative trait loci (QTLs) were highly-associated (P ≤ 5 × 10^−7^) with the multifocal (heritability = 0.075) and the diffuse (heritability = 0.189) lesions, respectively. No overlap was seen in the SNPs controlling these distinct pathological outcomes. The identified QTLs overlapped with some QTLs previously associated with PTB susceptibility, bovine tuberculosis susceptibility, clinical mastitis, somatic cell score, bovine respiratory disease susceptibility, tick resistance, IgG level, and length of productive life. Pathway analysis with candidate genes overlapping the identified QTLs revealed a significant enrichment of the keratinization pathway and cholesterol metabolism in the animals with multifocal and diffuse lesions, respectively. To test whether the enrichment of SNP variants in candidate genes involved in the cholesterol metabolism was associated with the diffuse lesions; the levels of total cholesterol were measured in plasma samples of cattle with focal, multifocal, or diffuse lesions or with no visible lesions. Our results showed reduced levels of plasma cholesterol in cattle with diffuse lesions. Taken together, our findings suggested that the variation in MAP-associated pathological outcomes might be, in part, genetically determined and indicative of distinct host responses.

## Introduction

Paratuberculosis (PTB) or Johne´s disease is a chronic enteritis of domestic and wild ruminants caused by *Mycobacterium avium* susbp. *paratuberculosis* (MAP). PTB is a major problem for animal health and must be notified to the World Organization for Animal Health. In Europe and North America, PTB is considered endemic in dairy cattle, with herd prevalence estimates higher than 50%^1^. PTB causes great economic losses to the dairy industry due to decreased milk production, weight loss, replacement cost, reduced slaughter value, a greater risk to other health problems, premature culling or death from the clinical disease, and the costs of veterinary expenses and control measures^2,3^. Infection occurs in the first months of life, primarily through the fecal–oral route, but clinical onset only takes place around calving when animals are 18 months or older. The most common clinical signs are decreased milk yield, chronic diarrhea, and progressive weight loss that eventually result in death of the animal^4^. However, most infected animals do not develop clinical disease, and microbiological and immunological diagnostic tests are not sensitive enough to identify them^5^. MAP has been postulated as a possible trigger factor in several autoimmune diseases in humans such as Crohn’s disease (CD)^6^, type I diabetes^7^, multiple sclerosis^8^, or rheumatoid arthritis^9^. It has been hypothesized that some antigenic structures of MAP are cross-reactive to self-proteins and might be responsible for these human autoimmune diseases in genetically predisposed individuals^10^. Colorectal cancer is a complication of the two forms of idiopathic inflammatory bowel disease (IBD); colonic CD and ulcerative colitis. Interestingly, MAP bacilli have been detected in the intestines of patients with CD, ulcerative colitis, and IBD-associated colorectal cancer^11,12^.

MAP causes lesions in naturally and experimentally infected cattle that differ in severity. According to their extension, cellular inflammatory infiltrate composition, and amount of MAP present in the lesions, PTB-associated lesions were classified into the following categories: focal, multifocal, and diffuse (diffuse paucibacillary or lymphoplasmacytic, diffuse intermediate, and diffuse multibacillary or histiocytic)^13,14^. Focal lesions consist of small granulomas in the ileal and jejunal lymph nodes or the ileocecal lymphoid tissue. In the multifocal lesions, middle size granulomas appear in the apex of some intestinal villi, as well as in the lymph nodes. These middle size granulomas are formed by groups of macrophages, surrounded by lymphocytes, and do not cause diffuse enteritis or modify the normal architecture of the intestine. In the diffuse paucibacillary or lymphoplasmacytic lesions, lymphocytes are the main cellular type, with some macrophages containing few if any mycobacteria. In diffuse intermediate lesions, the infiltrate is formed by abundant lymphocytes, macrophages, and Langhans giant cells with moderate numbers of acid-fast bacilli. Diffuse multibacillary or histiocytic lesions associate with severe granulomatous enteritis affecting different intestinal locations and lymph nodes, and are formed mainly by foamy macrophages loaded with cholesterol and large numbers of MAP. Two disease phenotypes, latent and patent, have been established with most infected cows classified as latent^5^. Clinical signs are exclusively associated with the diffuse lesions.

There is considerable variation among individuals in the response to MAP infection, a proportion of which is genetic. In a previous study, we compared the genetic determinants associated with ante-mortem (serum ELISA) and post-mortem (tissue PCR and culture) diagnostic definitions in a common set of animals using whole-genome sequence (WGS) data^15^. Combination test interpretation (all tests negative equals non-infected and all tests positive equals infected) was used to increase the sensitivity of the ELISA-Tissue PCR-tissue culture combination. This strategy reduced the risk of misclassification and increased the hereditability (h^2^) of the trait (h^2^ = 0.139) when compared with the h^2^ of the individual tests (h^2^ < 0.08). In the current study, we explored the genetic basis of the PTB-associated pathology and whether the post-mortem examination of gut tissues and regional lymph nodes could improve the accuracy of the classification of infected animals and uninfected controls providing higher h^2^ estimates. For this purpose, variance components and h^2^ were estimated for each type of MAP-associated histopathological lesion in a population of Spanish Holstein cattle (N = 813). Subsequently, single nucleotide polymorphisms (SNPs), quantitative trait loci (QTLs), candidate genes within significant QTLs, and functional pathways were identified.

## Results

### Variance components and h^2^ estimates

Initially, the h^2^ estimates were calculated for the presence (N = 440) or absence (N = 373) of any type of histopathological lesion in the case–control population. When contrasting these two PTB outcomes, the h^2^ estimate was 0.155 but no SNPs surpassed the FDR < 0.05. Consequently, h^2^ estimates were calculated for the presence or absence of each specific type of histopathological lesion; ranging from 0.075 for the multifocal lesions, 0.145 for the focal, and 0.189 for the diffuse lesions. The variance components, h^2^ estimates, and frequency of cases with focal (49.87%), multifocal (8.13%) or diffuse lesions (8.80%) are presented in Table 1. Control cows did not have visible lesions in gut tissues and had a negative ELISA, tissue PCR and culture result at the time of slaughter.

**Table 1:** Number of cases and controls, variance components, standard errors (SE), and h^2^ estimates.

PTB-associated lesion	Cases (%)	Controls (%)	Num. of SNPs Moderate association	Num. of SNPs High association	Additive genetic variance (σ^2^_G_)	SE	Residual variance (σ^2^_e_)	SE	Heritability (h^2^)
Focal	371 (49.87)	373 (50.13)	0	0	0.036345	0.019569	0.213189	0.021248	0.145651
Multifocal	33 (8.13)	373 (91.87)	277	129	0.005637	0.010162	0.069210	0.011124	0.075320
Diffuse	36 (8.80)	373 (91.20)	647	92	0.014969	0.009401	0.063845	0.009981	0.189929

### SNPs, QTLs, and candidate genes identification

**Figure 1:**
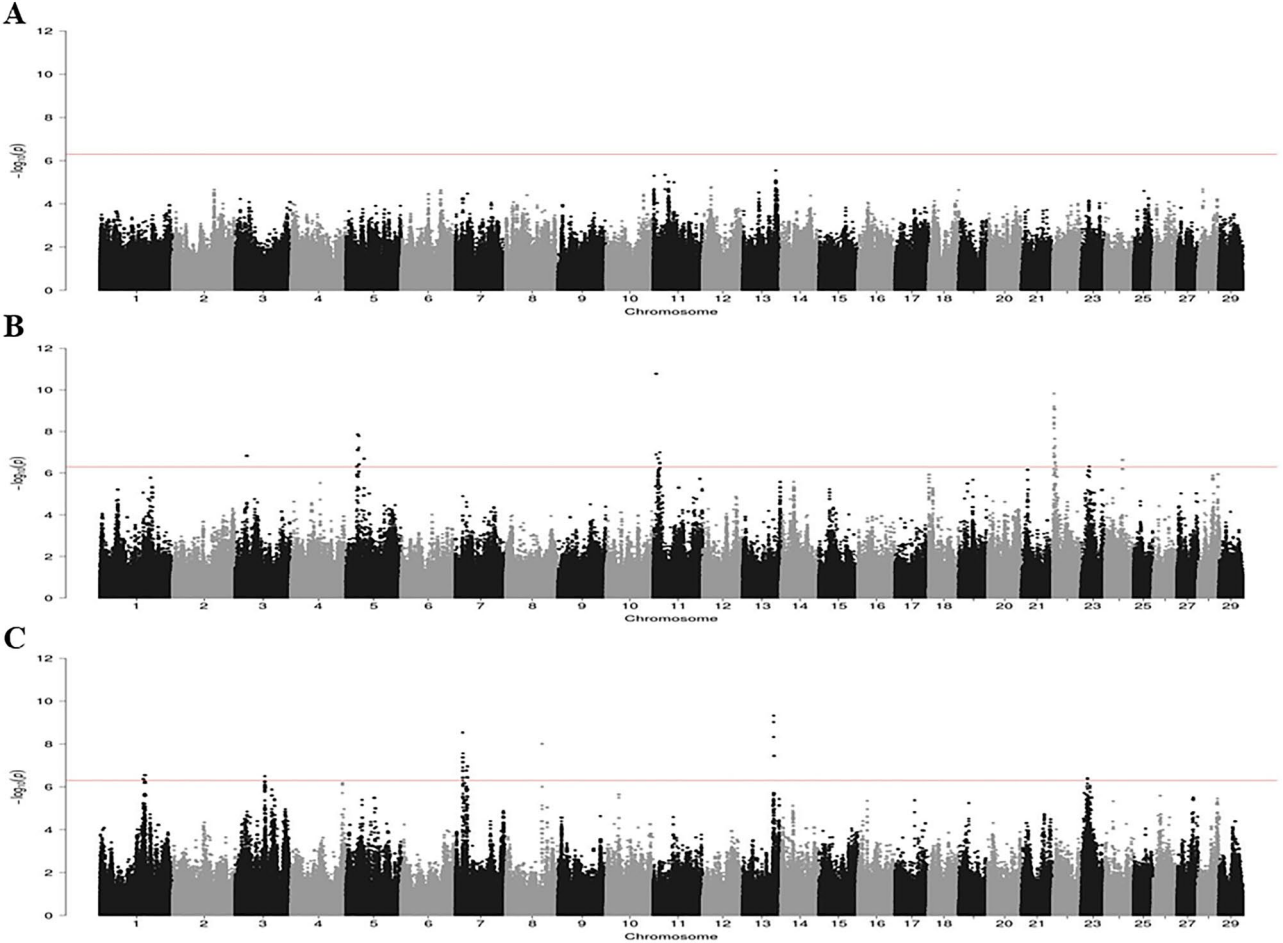
Manhattan plots showing –log_10_(*P* values) of association between every single SNP and phenotype. Each dot represents the result from the test association for a single SNP. Animals were considered cases when they had focal (**A**), multifocal (**B**) or diffuse (**C**) lesions. Chromosomes localization of the SNPs associated with each type of lesion is indicated on the x-axis. The red horizontal line is drawn at − log_10_ (5 × 10^−7^) to show the high level of significance.

To explore the genetic basis of the PTB-associated pathology, a genome-wide association study (GWAS) using the WGS datasets and the presence or absence of focal, multifocal or diffuse lesions was performed. The number of SNPs that surpassed the moderate (between P = 5 × 10^−5^ and P = 5 × 10^−7^) and the high (P < 5 × 10^−7^) thresholds is presented in Table 1. A total of 277 and 647 SNPs were moderately-associated with the multifocal and diffuse lesions, respectively. With the high threshold of association, we identified 129 and 92 SNPs highly-associated with the multifocal and the diffuse lesions, respectively. Interestingly, no overlap was seen in the SNPs with high and moderate association with the multifocal and diffuse lesions. As seen in Fig. 1A, there was not any SNP surpassing the high threshold of association with the focal lesions. In contrast, several chromosomal regions were highly-associated with the multifocal (Fig. 1B) and diffuse (Fig. 1C) lesions. While SNPs highly-associated with the multifocal lesions were located in BTA3, BTA5, BTA11, BTA22, BTA23, and BTA24, associations whit the diffuse lesions were found in BTA1, BTA3, BTA7, BTA8, BTA13 and BTA23. For the multifocal and diffuse lesions, most of the highly-associated SNPs were located in intronic regions; 50 and 51%, respectively. For the multifocal lesions, 18 and 12% of the SNP were downstream and upstream gene variants, respectively. In contrast, only 2 and 5% of the SNPs associated with the diffuse lesions were downstream and upstream gene variants and none of the diffuse-associated SNPs were in 3´UTR positions (Supplementary Fig. 1).

A description of the SNPs associated with the multifocal lesions surpassing the threshold (P < 5 × 10^−7^), *P* values, along with candidate genes located within the defined QTLs are presented in Table 2. The 129 SNPs associated with the multifocal lesions resided within 13 QTLs on 6 chromosomes including BTA3, BTA5, BTA11, BTA22, BTA23, and BTA24. The BTA5 harbored 4 of the 13 QTL regions. The quantitative trait locus (QTL) that harbored the most genome-wide significantly-associated SNP was located on BTA11 (5.22–6.61 Mb). The largest QTL was located on BTA22 (2.63–4.93 Mb). By examining the available cattle QTL database, we observed that the QTL that harbored the most genome-wide significantly-associated SNP (rs438855113) located on BTA11 (5.22–6.61 Mb) overlapped with regions previously associated with susceptibility to PTB (QTL169911, QTL169912, and QTL169913)^16^, clinical mastitis (QTL5450)^17^, and tick resistance (QTL101152 and QTL101166)^18^ (Supplementary Table 1). Another QTL associated to the multifocal lesions and located in BTA5 (27.26–28.35 Mb) overlapped with a QTL previously associated to PTB susceptibility (QTL14844)^19^. Interestingly, the QTL identified in BTA23 (17.32–18.32) overlapped with QTLs associated with blood IgG levels (QTL66214, QTL66215, QTL66217, QTL66218, QTL66219, QTL66220, QTL66221, QTL66222, QTL20493)^20^. Genes within this region such as the *NFKB Inhibitor Epsilon* (*NFKBIE*) control the response to several bacterial and viral pathogens as well as vaccines, possibly by influencing antibody production. The candidate genes associated with the multifocal lesions are novel in the sense that they have not been associated with PTB risk in cattle before except the *Neuronal PAS domain protein 2* (*NPAS2)* a member of the basic helix-loop-helix (bHLH)-PAS family of transcription factors. *NPAS2* is a core component of the circadian clock, an important regulator of a wide array of physiological functions including metabolism, sleep, body temperature, blood pressure, endocrine, immune, cardiovascular, and renal function.

**Table 2:** QTLs surpassing the significance threshold (P < 5 × 10^−7^) for evidence of an association with the multifocal lesions.

BTA^1^	QTL start (bp)	QTL end (bp)	*P* value most significant SNP	SNP position^2^	Annotation	Genes in QTL^3^	No of significant SNPs in QTL
3	24233907	25325344	1.50E−07	24825344	Intergenic	*TBX15, U6, SPAG17, GDAP2, WDR3, ENSBTAG00000048491, ENSBTAG00000051931*	12
5	24941743	26254968	1.39E−08	25667192	Intron	*VEZT, METAP2, USP44, MUCL1, GLYCAM1, PPP1R1A, PDE1B, NCKAP1L, ZNF385A, GTSF1, GPR84, ****ITGA5****, COPZ1, NFE2, HNRNPA1, CBX5, SMUG1, HOXC5, HOXC4, HOXC6, HOXC13, HOXC8, HOXC9, HOXC10, HOXC11, HOXC12, U6, ENSBTAG00000052805, ENSBTAG00000053941, ENSBTAG00000029988, ENSBTAG00000049200, ENSBTAG00000023670 ,ENSBTAG00000049405, ENSBTAG00000010711, ENSBTAG00000053336, ENSBTAG00000049915, ENSBTAG00000049837, ENSBTAG00000048717, ENSBTAG00000052958, ENSBTAG00000055223, ENSBTAG0000005385, ENSBTAG00000036381, ENSBTAG00000053232, ENSBTAG00000029788, ENSBTAG00000053186, ENSBTAG00000052715, ENSBTAG00000052501, ENSBTAG00000051607, ENSBTAG00000051555*	24
5	38788039	39790008	2.04E−07	39288039	Intron	***PDZRN4****, CNTN1, ENSBTAG00000001079*	2
5	23274617	24292059	4.92E−07	23774617	Intergenic	*UBE2N, MRPL42, SOCS2, CRADD, PLXNC1, CEP83, TMCC3, ENSBTAG00000033531, ENSBTAG00000049886, ENSBTAG00000053589, ENSBTAG00000050045, ENSBTAG00000052916*	5
5	27260957	28354826	1.69E−08	27777338	Intergenic	*KRT77, KRT1, KRT2, KRT73, KRT72, KRT74, KRT5, KRT6A, KRT6B, KRT75, KRT82, KRT84, KRT85, KRT89, KRT83, KRT81, KRT7, KRT80, SMIM41, ATG101, NR4A1, TAMALIN, ACVR1B, ACVRL1, ANKRD33, FIGNL2, SCN8A, U6, SLC4A8, ENSBTAG00000054953, ENSBTAG00000040019, ENSBTAG00000052798, ENSBTAG00000054816, ENSBTAG00000054136, ENSBTAG00000016166, ENSBTAG00000049194, ENSBTAG00000023471*	6
11	12914642	14422761	1.01E−07	13611793	Intron	*DYSF, ZNF638, PAIP2B, ATP6V1B1, ****FIGLA****, NAGK, VAX2, CLEC4F, TEX261, CD207, ANKRD53, ADD2, TGFA, FAM136A, XDH, SRD5A2, ENSBTAG00000048791, ENSBTAG00000051968, ENSBTAG00000050557, ENSBTAG00000051045*	4
11	5223771	6618872	1.66E−11	5755689	Intergenic	*AFF3, CHST10, NMS, PDCL3, NPAS2, TBC1D8, CNOT11, RFX8, RNF149, CREG2, MAP4K4, ENSBTAG00000050215, ENSBTAG00000043158, ENSBTAG00000054755*	7
11	9416584	10698629	1.98E−07	9916584	Intergenic	*TACR1, POLE4, U6, HK2, SEMA4F, M1AP, RTKN, DOK1, MRPL53, LOXL3, WBP1, DCTN1, HTRA2, WDR54, AUP1, DOX1, C11H2orf81, MGC152281, TLX2, PCGF1, LBX2, CCDC142, MOGS, INO80B, SLC4A5, MOB1A, MTHFD2, BOLA3, TET3, DGUOK, ENSBTAG00000049427 , ENSBTAG00000050345, ENSBTAG00000010072, ENSBTAG00000050408, ENSBTAG00000049783*	3
22	5865513	6865513	4.85E−07	6365513	Intron	*STT3B, ****OSBPL10****, GPD1L, CMTM8, CMTM7, ENSBTAG00000050895*	1
22	2638700	4937859	8.62E−10	3138700	Intergenic	*CMC1, AZI2, RBMS3, bta-mir-11990, U6, ENSBTAG00000022751, ENSBTAG00000050982, ENSBTAG00000048601, ENSBTAG00000052346*	15
22	1280377	2574848	1.50E−10	2008232	Intergenic	*U1, SEC61G, NEK10, U6, SLC4A7, 5S_rRNA, EOMES*	18
23	17326599	18326599	4.92E−07	17826599	Intergenic	*VEGFA, U6, MYMX, SPATS1, TMEM63B, HSP90AB1, CDC5L, CAPN11, NFKBIE, SLC9A1, SLC35B2, TMEM151B, AARS2, SUPT3H, 5S-rRNA, ENSBTAG00000050989, ENSBTAG00000049965, ENSBTAG00000050049, ENSBTAG00000050249, ENSBTAG00000050117, ENSBTAG00000053551, ENSBTAG00000053897*	1
24	37183179	38191028	2.33E−07	37683179	Intron	*EMILIN2, LPIN2, MYOM1, MYL12A, MYL12B, TGIF1, ****DLGAP1****, SNORA70, ENSBTAG00000052283*	31

^1^QTL location, ^2^SNP location in the genome assembly, ^3^Positional candidate genes are defined as genes that are located within 50 kb on either side of the identified QTL.

The GWAS defined 92 SNPs and 9 QTLs with a high association with the diffuse lesions located in 6 chromosomes (BTA1, BTA3, BTA7, BTA8, BTA13 and BTA23) (Table 3). By examining the available cattle QTL database, we observed that a QTL located on BTA7 (23.79–25.08 Mb) overlapped with a QTL previously associated with PTB susceptibility (QTL166688) (Supplementary Table 2)^21^. The QTL on BTA3 (63.43–64.43 Mb) overlapped with the QTL167791 and QTL2489 associated with bovine tuberculosis (bTb) susceptibility and clinical mastitis, respectively^22,23^. In addition, the QTL identified in BTA23 (13.56–14.56 Mb) overlapped with QTLs associated with bTb (QTL96552), clinical mastitis (QTL164913), and IgG levels (QTL66214, QTL66215, QTL66216, QTL66217, QTL66218, QTL66219, QTL66220, QTL66221, and QTL66222)^20,24,25^. The candidate genes associated with the diffuse lesions had not been associated with PTB risk in cattle before except the *Dendritic Cell-Specific Intracellular Adhesion Molecules (ICAM)-3* or *CD209*^26,27^.

**Table 3:** Quantitative trait loci (QTL) surpassing the significance threshold (P < 5 × 10^−7^) for evidence of an association with the diffuse lesions.

BTA^1^	QTL start (bp)	QTL end (bp)	*P* value most significant SNP	SNP position^2^	Annotation	Genes in QTL^3^	No of significant SNPs in QTL
1	97006475	98111140	2.78E−07	97523460	Intergenic	*SLC7A14, CLDN11, SKIL, PRKCI, U2, PHC3, U6, GPR160, SEC62, SAMD7, LRRIQ4, LRRC34, MYNN, ACTRT3, Telomerase-vert, ENSBTAG00000049676, ENSBTAG00000042535, ENSBTAG00000045991, ENSBTAG00000053891, ENSBTAG00000047144*	35
1	93955885	94925704	4.30E−07	94425704	Intron	***SPATA16,**** ECT2, NCEH1*	8
3	63439078	64439078	3.16E−07	63939078	Upstream	*U6****,**** ENSBTAG00000052602*	1
7	15613384	17275615	2.91E−09	16113384	Intron	*PDE4A, KEAP1, s1PR5, ATG4D, KRI1, CDKN2D, AP1M2, SLC44A2, ILF3, QTRT1, DNM2, TMED1, C7H19orf38, CARM1, bta-mir3604-1, YIPF2, SMARCA4, TIMM29, TMED1, SPC24, KANK2,5S_rRNA, U6, LDLR, RAB3D, TMEM205, CCDC159, DOCK6, ANGPTL8, ELAVL3, ELOF1, PLPPR2, ACP5, EPOR, RGL3, ODAD3, PRKCSH, ZNF653, ECSIT, U4, ****INSR****, ZNF557, ARHGEF18, CAMSAP3, PEX11G, RETN, XAB2, TEX45, MCEMP1, PET100, ZNF358, MCOLN1, PNPLA6, PCP2, STXBP2, TRAPPC5, FCER2, LRRC8B, CD209, MAP2K7, CLEC4G, EVI5L, CCL25, SNAPC2, TIMM44, bta-mir-2455, ELAVL1, FBN3, MARCHF2, CERS4, HNRNPM, ADAMTS10, bta-mir-339b, ANGPTL4, ACTL9, CD320, PRAM1, NDUFA7, ZNF414, RAB11B, OR2Z1, RPS28, MYO1F, SNORA70, ENSBTAG00000051140, ENSBTAG00000051490, ENSBTAG00000054591, ENSBTAG00000046336, ENSBTAG00000038680, ENSBTAG00000047612, ENSBTAG00000044018, ENSBTAG00000052882, ENSBTAG00000049668, ENSBTAG00000054873*	21
7	26041927	27059884	1.12E−07	26547965	Intergenic	*CCDC192, 5S_rRNA, bta-mir-12007, CTXN3, PRRC1, MEGF10, U6, C7H5orf63, MARCHF3, ENSBTAG00000051246*	12
7	23796173	25086968	1.73E−07	24296173	Intergenic	*CHSY3, MINAR2, ADAMTS19, ISOC1, SLC27A6, ENSBTAG00000051108, ENSBTAG00000051190*	4
8	77894983	78394983	9.77E−09	78394983	Intergenic	*NTRK2*	2
13	66829928	68799183	4.70E−10	67404014	Intron	*VSTM2L, TTI1, RPRD1B, TGM2, U6, LIAA1755, BPI, LBP, SNORA71, ****RALGAPB****, ADIG, SLC32A1, ACTR5, PPP1R16B, FAM83D, DHX35, ENSBTAG00000051355, ENSBTAG00000047302, ENSBTAG00000050770, ENSBTAG00000052551, ENSBTAG00000044690*	6
23	13569222	14569289	3.91091E−07	14069222	Intergenic	*KIF6, DAAM2, MOCS1, U6, LRFN2, ENSBTAG00000048842*	3

^1^QTL location, ^2^SNP location in the genome assembly, ^3^Positional candidate genes are defined as genes that are located within 50 kb on either side of the identified QTL.

### Quantile–quantile plots and odds ratios

Quantile–quantile plots comparing the observed distribution of –log (*P* values) to the expected values under null hypothesis were generated. The plots showed a distribution close to the expected distribution for the following phenotypes: focal vs undetected lesions (λ_median_ = 1.01689), multifocal vs undetected lesions (λ_median_ = 1.04401), and diffuse versus undetected lesions (λ_median_ = 1.03222), indicating that significant values were not overestimated due to population stratification or cryptic relatedness. A slight deviation in the upper right tails from the y = x line suggested that some associations were present in the data. The regression coefficients (b-values) for all the SNPs associated with the presence of multifocal and diffuse lesions were all positive suggesting a positive correlation between exposure and outcome (data not shown). In addition, the odds ratios (OR) calculated for all the SNPs associated with the presence of multifocal and diffuse lesions (P < 5 × 10^−7^) were > 1 indicating that the animals with the minor alleles had a higher risk of developing multifocal or diffuse lesions (Supplementary Table 3).

### Gene ontologies and metabolic pathways

Functional categorization of the candidate genes for each phenotype (presence or absence of multifocal and diffuse lesions) was performed using the Bioconductor GOseq package. We identified 7 GO and 1 metabolic pathway significantly enriched in the animals with multifocal and diffuse lesions, respectively. As showed in Table 4, seven cellular components (CC) related to the intermediate filament cytoskeleton were significantly enriched in the multifocal lesions. Gene members of the *Keratin* (*KRT*) family such as the *KRT5, KRT7, KRT72, KRT73, KRT74, KRT75, KRT80, KRT81,* and *KRT83* were common to these seven CC. In the diffuse lesions, the cholesterol metabolism (bta04979) was enriched (*P* adjust = 0.016) with 4 candidate proteins matching this route including the *Angiopoietin like 4 and 8 (ANGPTL4* and *ANGPTL8*), *Low Density Lipoprotein Receptor* (*LDRL*), and *Neutral Cholesterol Ester Hydrolase 1* (*NCEH1).* The *ANGPTL*4 and *ANGPTL8* play a role in the regulation of triglyceride clearance from the serum and in lipid metabolism. The *LDRL* binds *LDL*, the major cholesterol-carrying lipoprotein of plasma, and transports it into cells, including macrophages, by endocytosis. The *NCEH1* is responsible for cholesterol ester hydrolysis in macrophages.

**Table 4:** GO and pathway analysis using the candidate genes associated with the multifocal and diffuse lesions.

Phenotype	ID	Description	*P* adjust	Genes	Genes ratio
Multifocal	GO:0045095	Keratin filament	6.816E−13	KRT73/KRT72/KRT74/KRT5/KRT75/KRT83/KRT81/KRT7/KRT80	9/44
	GO:0005882	Intermediate filament	1.691E−10	KRT73/KRT72/KRT74/KRT5/KRT6B/KRT75/KRT83/KRT81/KRT7/KRT80	10/44
	GO:0045111	Intermediate filament cytoskeleton	4.644E−10	KRT73/KRT72/KRT74/KRT5/KRT6B/KRT75/KRT83/KRT81/KRT7/KRT80	10/44
	GO:0099512	Supramolecular fiber	4.389E−07	KRT73/KRT72/KRT74/KRT5/KRT6B/KRT75/KRT83/KRT81/KRT7/KRT80/MYOM1/MYL12A/MYL12B	13/44
	GO:0099081	Supramolecular polymer	4.389E−07	KRT73/KRT72/KRT74/KRT5/KRT6B/KRT75/KRT83/KRT81/KRT7/KRT80/MYOM1/MYL12A/MYL12B	13/44
	GO:0099513	Polymeric cytoskeletal fiber	1.689E−05	KRT73/KRT72/KRT74/KRT5/KRT6B/KRT75/KRT83/KRT81/KRT7/KRT80	10/44
	GO:0099080	Supramolecular complex	2.1E−05	KRT73/KRT72/KRT74/KRT5/KRT6B/KRT75/KRT83/KRT81/KRT7/KRT80/MYOM1/MYL12A/MYL12B	13/44
Diffuse	bta04979	Cholesterol metabolism	0.016505	NCEH1/LDLR/ANGPTL8/ANGPTL4	4/49

### Antikeratin antibodies (AKA) and cholesterol plasma levels

**Figure 2:**
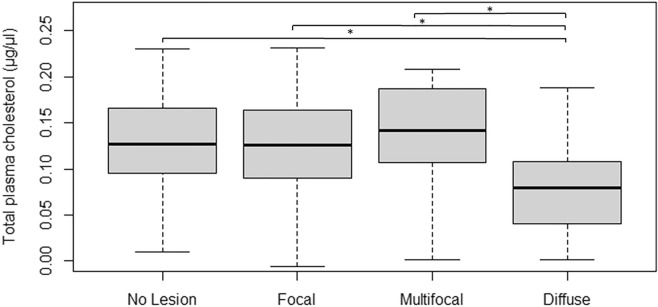
Comparison of plasma cholesterol of controls and cows with focal, multifocal or diffuse lesiosn. Samples were selected from the case–control population and grouped according to the presence or absence of visible lesions in gut tissues. Cows with diffuse lesions (N = 26) were associated with decreased cholesterol plasma levels when compared with no visible lesions (N = 474) or with focal (N = 350) and multifocal (N = 32) lesions. The lines extending from the boxes indicate variability outside the upper and lower quartiles. The highest and lowest points are the maximum and the minimum of the data set, respectively. *P* values were calculated using an unpaired t-test. *P value < 0.001.

To test whether a positive correlation between the levels of AKA in plasma and the presence of multifocal lesions existed, AKA levels were measured in plasma samples of cows with multifocal lesions and without visible lesions. No significant differences (*P* = 0.2) were found between the levels of AKA in cows with multifocal lesions (1.44 ng/ml) in comparison with cows with no visible lesions (2.10 ng/ml). As presented in Table 4, enrichment of candidate genes controlling the cholesterol metabolism (bta04979) was identified in cows with diffuse lesions. To test whether the enrichment of SNP variants in candidate genes caused dysregulation of the cholesterol metabolism, the levels of total cholesterol were measured in plasma samples from cattle with focal, multifocal or diffuse lesions or with no visible lesions. As seen in Fig. 2, there was a significant decrease in total cholesterol in plasma samples from cows with diffuse lesions (0.080 µg/µl) compared to those with focal (0.126 µg/µl; *P* ≤ 0.001), or multifocal (0.141 µg/µl, *P* ≤ 0.001) lesions, or with no visible lesions (0.129 µg/µl, *P* ≤ 0.001).

## Discussion

PTB is a multifactorial disease that arises as the result of the interaction of genetic, environmental, and microbial factors leading to the various PTB outcomes. Genetic factors are likely to play an important role in PTB pathogenesis. Cattle infected with MAP display various types of lesions with distinct severity but the associations between host genetic and PTB-associated pathology had not been explored before. Although previous GWAS identified loci associated with MAP tissue infection assessed by PCR and culture^15,28–30^, our study provides the first comparison of the genetic variants associated with three distinct PTB-associated lesions (focal, multifocal and diffuse) in Spanish Holstein cattle (N = 813). Our study is unique in the definition of cases and controls through the histopathological analysis of PTB-associated lesions. Using WGS data, the h^2^ estimates were calculated for the presence or absence of PTB-associated histopathological lesions in Spanish Holstein cattle (N = 813). When contrasting these two PTB outcomes, we couldn´t identify any SNP surpassing the FDR < 0.05. However, the stratification of PTB-associated pathology in three categories allowed the identification of SNPs surpassing the FDR < 0.05 and increased the h^2^ of the diffuse lesions (h^2^ = 0.189) when compared with phenotypes such as the ELISA-tissue PCR-tissue culture (h^2^ = 0.139) that typically detect animals with diffuse lesions^15^. The h^2^ estimates calculated for the focal (h^2^ = 0.145), multifocal (h^2^ = 0.075), and diffuse (h^2^ = 0.189) lesions indicated that the PTB-associated pathological outcomes have a genetic component that consists of a large number of genetic variants located across many chromosomes of the bovine genome. Previous studies indicated that susceptibility to MAP infection is heritable, with h^2^ estimates of susceptibility to the infection ranging from < 0.01^29,30^ to 0.2843^31^. This is comparable with the moderate heritability (h^2^ = 0.12) estimated for bTb infection even though the phenotype definitions and models used differed^22^. In a previous study, Wilkinson et al., genotyped 1966 Holstein–Friesian dairy cows following *Mycobacterium bovis* infection with three distinct phenotypes: single intradermal cervical comparative tuberculin (SICCT) test positives with visible lesions, SICCT-positives with undetected visible lesions, and SICCT-negative on multiple tests^32^. Regardless of the case phenotype, h^2^ estimates did not exceed h^2^ = 0.08. Although the h^2^ of clinical mastitis in dairy cattle is also moderate (0.07–0.11)^33^, the somatic cell score is included in selective breeding programs in many countries, including Spain.

The GWAS did not identify any SNP associated with the focal lesions. This could be attributed to the lack of discrimination between no visible and focal lesions. Using RNA-Seq technology we previously identified a biomarker of PTB progression, the precursor of the bovine intelectin 2 (ITLN2), which was overexpressed in ICV samples of animals with focal (log_2_ fold-change = 10.6) and diffuse (log_2_ fold-change = 6.8) lesions compared with animals without visible lesions^34^. More recently, we have demonstrated that the quantification of bovine ITLN2 secreting cells by immunohistochemical analysis of ICV sections could constitute a good post-mortem tool, complementary to the histopathology, to improve the detection of focal lesions which may sometimes be difficult to detect^35^. To test whether the focal lesions were controlled by specific genetic variants, a GWAS comparing animals with focal versus multifocal and focal versus diffuse lesions was performed (data not shown). A total of 28 and 590 SNPs were specifically associated with each comparison (FDR < 0.05), respectively. These results revealed significant differences in the genetic variants associated with the presence of focal lesions when compared with the multifocal or diffuse lesions. Further GWAS using diagnostic methods allowing a better discrimination of the animals with focal and no visible lesions are needed.

A total of 192 and 92 SNPs defining 13 and 9 distinct quantitative trait loci (QTLs) were highly-associated with the multifocal and diffuse lesions, respectively. All these SNPs had a positive b-value and OR > 1 and were, therefore, associated with each pathological outcome. No overlap was seen in the SNPs associated with each type of lesion which suggested that distinct genetic variants might control the multifocal and diffuses lesions and that these lesions might represent divergent disease outcomes. Further blinded studies are required to validate the associations between the identified SNPs and corresponding pathological outcomes in an independent population. Although the functional effects of the identified candidate genes were assigned through GO and KEGG pathways, further functional studies need to be performed on selective SNPs to determine if they are affecting the positional candidate genes or other genes through regulatory effects. Pathway analysis with candidate genes overlapping the lesions-associated QTLs revealed a significant enrichment of variants controlling the intermediate filament cytoskeleton and cholesterol metabolism in the animals with multifocal and diffuse lesions, respectively. Our findings provide for the first time a potential link between genetic variants in *KRT* genes (*KRT5, KRT7, KRT72, KRT73, KRT74, KRT75, KRT80, KRT81,* and *KRT83*) and PTB. Interestingly, abnormal *KRT* mutations have been associated with IBD^36^. It was previously reported the sharing of mimicking B cell epitopes between *M. leprae* and the host *KRT7*^37^. Similarly, we hypothesized that molecular mimicry between putative epitopes of *KRT* and MAP might be playing a role in the development of PTB-associated multifocal lesions. However, no correlation between high levels of AKA in plasma and cows with multifocal lesions was observed. In the other hand, *KRTs*, the major subgroup among the intermediate filament family of cytoskeletal proteins, are responsible for maintaining the stability and integrity of the gastrointestinal epithelium, for providing tissue resilience against many stimuli, and regulating various cellular functions such as cellular proliferation, migration, differentiation as well as inflammatory and immune responses^38^. In a granulomatous experimental model using the teleost fish *Piaractus mesopotamicus* infected with Bacillus Calmette–Guérin (BCG), stronger immunostaining with anti-cytokeratin antibodies was observed at 33 days p. i. when the epithelioid cells were more evident and the granulomas were fully formed^39^. In tuberculoid (TT) and borderline tuberculoid leprosy, epithelioid non-caseinated granulomas encapsulated by many lymphocytes predominate and acid-fast bacilli are absent or only rarely present^40^. The presence of epithelioid granulomas with multifocal distribution in TT leprosy leads to the control of *M. leprae* replication and the containment of its spread^41^. Further immunohistochemical studies with a bovine anti-cytokeratin antibody are needed to quantify the number of *KRT*-stained cells in PTB-associated lesions and to reveal the potential role of *KRT* in maintaining tissue resilience in animals with multifocal lesions.

While *KRT* variants were associated with cows with multifocal lesions, genetic variants in candidate genes involved in the cholesterol metabolism were enriched in animals with diffuse lesions, thereby suggesting that cholesterol variants associated with disease progression. To test whether the enrichment of SNP variants in candidate genes (*NCEH1/LDLR/ANGPTL8/ANGPTL4*) involved in the cholesterol metabolism was associated with the diffuse lesions, the levels of total cholesterol were measured in plasma samples of cattle with focal, multifocal or diffuse lesions or with no visible lesions. Our results showed reduced levels of plasma cholesterol in cattle with diffuse lesions (*P* ≤ 0.001). Similarly, inflammation has been associated with decreased total serum cholesterol levels in patients diagnosed with CD^42^ and IBD^43^. This reduction might be due to impedance of absorption of cholesterol efficiently thought the thickened intestinal wall in individuals in advances stages of the infection. Using RNA-Seq, we previously observed that the cholesterol route (bta04977) was dysregulated in the ileocecal valve (ICV) of cows with diffuse lesions versus the control group with four upregulated genes matching this route (*APOA1, APOC3, APOA4, APOB*)^34^. In addition, the top upregulated gene in peripheral blood of animals with focal lesions versus control cows was the *ATP-Binding Cassette Subfamily A Member 13* (*ABCA13*), a gene that accelerates cholesterol internalization and accumulation in intracellular vesicles^34,44,45^. In agreement with our data, recent evidence suggests that MAP-infected macrophages accumulate intracellular cholesterol droplets and depict a foam cell phenotype during infection providing an enriched environment for MAP survival^46,47^. Altogether, these findings suggest increased cholesterol transport, internalization and hydrolysis in macrophages of PTB-infected animals with diffuse lesions, which may invoke a localized compensatory mechanism to increase cholesterol synthesis at the site of the infection. It is well known that one of the main clinical signs associated to clinical PTB is decreased milk production. Animals with diffuse lesions have lower milk production and milk fat content when compared with animals with other lesions and uninfected cows^48^. If an animal shows decreasing plasma cholesterol levels that may be considered an indication that it is likely to have decreased milk production and progressing toward clinical disease.

## Conclusions

While PTB-associated multifocal lesions lead to a localized disease, the diffuse lesions present a disseminated form with high bacterial loads. Our results suggested that the genetic variants associated with the presence of focal, multifocal and diffuse lesions were distinct. While keratin variants associated with the multifocal lesions, cholesterol variants associated with the diffuse, more severe lesions. It follows that there are at least two distinct disease outcomes, which might be indicative of different host responses. Rearrangements in the keratin filaments and cholesterol metabolism are predominantly gearing this disease outcome polarization.

## Materials and methods

### Ethics statement

Animals used in this study were not submitted to any in vivo experimentation before stunning for slaughter and, therefore, no specific ethics committee authorization was required. The cows were slaughtered in the Bilbao and Donostia municipal slaughterhouses (Basque Country, Spain) under the pertinent Basque (Basque Government Decree 454/1994), Spanish (Spanish Government Law 32/2007 and Royal decree 731/2007), and European (Council Regulation (EC) No 1099/2009) legislation on animal welfare.

### Animals

The Spanish Holstein population included in this study consisted of 813 culled Holstein cattle from several herds located in eight regions: Basque Country (N = 212, 26.08%), Catalonia (N = 318, 39.11%), Navarre (N = 170, 20.91%), Cantabria (N = 59, 7.26%), Aragon (N = 24, 2.95%), Castile and Leon (N = 21, 2.58%), La Rioja (N = 6, 0.74%) and Asturias (N = 3, 0.37%). Only cows (18 months years or older, 5.5 years mean age) were included in the analyses as PTB has a long incubation period and older animals are, therefore, more likely to show clinical signs. The cows were slaughtered in two abattoirs located in the Basque Country from March 2007 to May 2010.

### Histopathological examination

Post-mortem tissue sampling was performed as previously described^49^. Briefly, samples from ileocecal lymph node, jejunal lymph node, ICV, jejunum, and terminal ileum were collected aseptically from each animal and placed in formalin within 24 h after arrival at the laboratory. The samples were preferentially taken from areas of the preselected tissues that showed gross lesions, thickened mucosa and enlarged lymphatic nodes, if present. The samples were fixed in 10% neutral buffered formalin for 72 h, dehydrated through graded alcohols and xilol, embedded in paraffin, and cut into 4 μm sections. Sections were, mounted on treated microscope slides, stained with haematoxylin and eosin (HE) and with Ziehl–Neelsen (ZN) and examined for pathological lesions and for the presence of acid-fast bacteria, respectively. According to their location and extension, inflammatory cell type, and mycobacterial load, PTB-associated histopathological lesions were classified in focal, multifocal, and diffuse lesions as previously described^13^.

### Genotyping and imputation to WGS

Peripheral blood samples were collected at the slaughter time and DNA was extracted using the QIAmp DNA Blood Mini Kit according to the manufacturer´s instructions (Qiagen, Hilden, Germany). Genotyping and imputation to WGS was performed as previously described^15^. Briefly, purified genomic DNA was quantified spectrophotometrically and subsequently genotyped with the EuroG MD Bead Chip at the molecular genetic laboratory service of the Spanish Federation of Holstein Cattle (CONAFE) using the InfiniumTM iScan software for allele assignation (Illumina, San Diego, CA). Individual genotypes were phased using Eagle 2.4^50^ and imputed with minimac4^51^ to the Bovine HD Bead Chip using a reference panel of 1,278 *Bos taurus* from Run7.0 of the 1000 Bull Genomes project and 581,712 SNPs (ASR-UCD1.2). Imputation to WGS level was then undertaken using a reference population of 2333 *Bos taurus* from Run6.0 of the 1,000 Bull Genomes project^52^. In total, 33.77 million SNPs per animal were identified across the genome. All the SNPs had a call rate > 0.80. PTB-associated SNPs with minimum allele frequency (MAF) < 0.01 were removed. The number of SNPs kept in the analysis was 13,881,067.

### Variance components and h^2^ estimates

The variance components, standard errors (SE), and h^2^ estimates explained by all the SNPs were calculated using the genome-wide complex trait analysis (GCTA) software 1.93.2, according to the following formula:$h^{2} = \frac{{\upsigma 2\;{\text{G}}}}{{\upsigma 2\;{\text{G}} + \upsigma 2\;{\text{e}}}}$ where σ_G_ is the additive genetic effect of the individuals and σ_e_ is the residual variance^53^. The variance components σ_G_ and σ_e_ in the equation were estimated by the genomic-relatedness-based restricted maximum-likelihood (GREML) approach implemented in GCTA1.93.2. The concept behind this method is to fit all the SNPs simultaneously as random effects in a mixed linear model to estimate the phenotypic variance explained by all the SNPs.

### Genome-wide association study

Associations between the imputed genotypes and the presence or absence of focal, multifocal or diffuse lesions was analyzed in a case–control study using the *mlma* (mixed linear model) association analysis of the GCTA 1.93.2^53^. Briefly, the model is *y* = *a* + *bx* + *g* + *e*, where *y* is the phenotype, *a* is the mean term, *b* is the additive effect (fixed effect) of the candidate SNP to be tested for association, *x* is the SNP genotype indicator variable coded as 0, 1 or 2, *g* is the polygenic effect (random effect) i.e. the accumulated effect of all SNPs (as captured by the GRM calculated using all SNPs), and *e* is the residual. Control cows did not have visible lesions in gut tissues and had a negative ELISA, tissue PCR and culture result at the time of slaughter. Age was included as a covariate in the analysis. After the GWAS, the SNPs with R^2^ values higher than 70% were retained. To account for multiple testing, a 5% chromosome-wise false discovery rate (FDR) was used to determine the probability that the associations were not false positives. *P* values between P = 5 × 10^−5^ and P = 5 × 10^−7^ provided a moderate significance level (α), and *P* values < 5 × 10^−7^ were used to identify highly significant associations (Welcome Trust Case Control Consortium, 2007). The inflation factor (λ) and quantile–quantile plots were calculated to compare observed distributions of –log (*P* values) to the expected distribution under the no association model for each phenotype. λ value close to 1 suggests appropriate adjustment for potential substructure and λ > 1.2 suggests population stratification. The regression coefficients (b) calculated using GCTA 1.93.2 represent the estimated increase in the log odds of the outcome per unit increase in the value of the exposure*.* In other words, the exponential function of b (*e*^*b*^) is the OR-associated with a one-unit increase in the exposure. In addition, the OR and their 95% confidence intervals (CI) for the SNPs associated with the presence or absence of each type of histopathological lesion (P < 5 × 10^−7^) were calculated using logistic regression analysis with the WGassociation function of SNPassoc 1.9.2 under five different genetic models (co-dominant, dominant, recessive, over-dominant, and log-additive)^54^. For each SNP and genetic model, the function WGstats of SNPassoc 1.9.2*.* provides genotype frequencies, OR, and 95% CI with the major homozygous genotype deemed as the baseline.

### SNPs, QTLs, and candidate genes identification

The location of the significant SNPs was determined with biomaRt 2.44.1 for R^55^ using the ARS-UCD1.2 reference genome. The genomic distribution of the identified SNPs was determined using the Ensembl Variant Effect Predictor (VEP). QTLs associated with each type of histological lesion were defined based on SNPs on linkage disequilibrium patterns with SNPs that surpassed the suggestive significance threshold (P < 5 × 10^−7^) in a given chromosome. The beginning and end of each QTL were defined in a window of 500,000 base pairs upstream and downstream by the SNPs that were furthest upstream and downstream of the suggestive SNP. Overlapping QTL regions were merged and considered as a single QTL. The defined QTL regions we further investigated for the presence of candidate genes within 50,000 base pairs to each side of the defined QTL using Ensembl (https://www.ensembl.org). The identified QTLs and candidate genes were compared with the reported cattle QTLs and candidate genes for animal diseases including PTB, bovine tuberculosis, clinical mastitis and bovine respiratory disease (http://www.animalgenome.org).

### Gene ontology and metabolic analysis

Candidate genes within significant QTLs were investigated for significant enrichments of GO categories and Kyoto Encyclopedia of Genes and Genomes (KEGG) pathways using the cluster Profiler Bioconductor package 3.10.1^56,57^. Briefly, the ClusterProfiler package offers a gene classification method, namely *groupGO*, to classify genes based on their projection at a specific level of the GO corpus, and provides functions, *enrichGO* and *enrichKEGG*, to calculate enrichment test for GO terms and KEGG pathways based on hypergeometric distribution. To prevent high false discovery rate (FDR) in multiple testing, *P* adjust values were estimated. GO analysis provides categories of genes involved in different biological processes (BP), molecular functions (MF), and those integral for different cell compartments (CC).

### Bovine anti-keratin antibody ELISA Kit

For the quantitative detection of AKA in plasma samples of animals without visible lesions (N = 33) and with multifocal lesions (N = 31), a double antigen sandwich ELISA kit was used following the manufacturer’s instructions (MyBiosource, US). Briefly, plasma samples (100 µl per well) were added into the ELISA plate pre-coated with a bovine AKA antigen and incubated at 37 °C for 90 min. Samples were washed out twice with PBS and then bovine AKA antigen (100 µl) was added. Plates were incubated at 37 °C form 60 min. ELISA plates were washed 3 times and an avidin-peroxidase conjugate (100 µl) was added to each well. The plates were sealed and incubated at 37 °C for 30 min. The enzyme conjugate was washed out of the wells five times with PBS and a TBM substrate (100 µl) was used for coloration. TBM reacts to form a blue product from the peroxidase activity, and finally turns to yellow after addition of 100 µl per well of the stop solution (Color Reagent C). Plates were read at 450 nm in an ELISA reader (Thermo Scientific Multiskan, US). The OD values of each sample and standard had the values of the blank subtracted. We averaged the duplicate OD readings for each standard and sample. A standard curve was generated by plotting the mean OD values of each standard on the vertical axis and the corresponding concentration on the horizontal axis. The AKA concentration levels in each sample were interpolated from the standard curve. Statistical analysis was performed using an unpaired t-test for comparison between two groups (*GraphPad Prism 8*, San Diego, California, US). Differences were considered significant when *P* ≤ 0.05.

### Cholesterol quantitation

A Cholesterol Quantitation kit (Sigma-Aldrich, St. Lois, USA) was used to determine the concentration of total cholesterol present in plasma samples of cows with no visible lesions (N = 474), and with focal (N = 350), multifocal (N = 32), and diffuse (N = 26) lesions. Total cholesterol concentration was determined by a coupled enzyme assay, containing cholesterol esterase, which results in a colorimetric product proportional to the cholesterol present. Cholesterol esterase hydrolyzes cholesteryl esters to cholesterol. Each reaction requires 2 µl of plasma which was brought to a final volume of 50 µl with cholesterol buffer. An equal volume (50 µl) of total cholesterol reaction master mix, containing cholesterol esterase, was added to each well and placed on a horizontal shaker for 5 min, after which the plate was incubated in the dark a 37 °C for 1 h. Following incubation, absorbance was measured at 570 nm using an ELISA reader (Thermo Scientific Multiskan, US). Cholesterol concentration (µg/µl) present in the plasma samples was calculated by comparison against a serial dilution of cholesterol standards provided with the kit. All samples and standards were run in duplicate. Statistical analysis was performed using an unpaired t-test for comparison between groups. Differences were considered statistically significate when *P* ≤ 0.05.

### Preprint

This article was submitted to an online preprint archive^58^.

## Supplementary Information

Supplementary Information.

## Data Availability

The sequence variants for 1800 animals from Run8 are public at the European Variation Archive under project number PRJEB42783. The SNPs highly-associated with MAP-associated pathology have been deposited in the animal genome database (https://www.animalgenome.org/). Individual genotype data used in this study is managed by a third party, the Spanish Friesian Cattle National Federation (CONAFE). Requests for genotype data can be made to CONAFE, Ctra. de Andalucía, km. 23,600–28340 Valdemoro, Madrid, Spain; email: conafe@conafe.com; phone: + 34 (91) 8952412; Fax: 918 951 471; website: www.conafe.com. CONAFE is a member of EuroGenomics Coöperative U.A.
