# Author Correction: Identification of loci associated with pathological outcomes in Holstein cattle infected with *Mycobacterium avium* subsp. *paratuberculosis* using whole-genome sequence data

**DOI:** 10.1038/s41598-021-02009-4

**Published:** 2021-11-16

**Authors:** Maria Canive, Gerard Badia-Bringué, Patricia Vázquez, Oscar González-Recio, Almudena Fernández, Joseba M. Garrido, Ramón A. Juste, Marta Alonso-Hearn

**Affiliations:** 1 Department of Animal Health, NEIKER-Basque Institute for Agricultural Research and Development, Basque Research and Technology Alliance (BRTA), Derio, Bizkaia Spain; 2 Doctoral Program in Immunology, Microbiology and Parasitology, Universidad del País Vasco/Euskal Herriko Unibertsitatea (UPV/EHU), Leioa, Bizkaia Spain; 3 Doctoral Program in Molecular Biology and Biomedicine, Universidad del País Vasco/Euskal Herriko Unibertsitatea (UPV/EHU), Leioa, Bizkaia Spain; 4 Departamento de Mejora Genética Animal, Instituto Nacional de Investigación y Tecnología Agraria y Alimentaria, CSIC, Madrid, Spain; 5 Departamento de Producción Agraria, Escuela Técnica Superior de Ingeniería Agronómica, Alimentaria y de Biosistemas, Universidad Politécnica de Madrid, Ciudad Universitaria, Madrid, Spain

Correction to: *Scientific Reports* 10.1038/s41598-021-99672-4, published online 11 October 2021

The Preprint section in the original version of this Article was omitted. The Preprint section now reads:

“This article was submitted to an online preprint archive^58^.”

As a result Reference 58 was omitted, which is listed below.

58. Canive, M., *et al*. Host Genetics Underlying Pathological Outcomes to *Mycobacterium Avium Subsp. Paratuberculosis* Infection is Governed by Distinct Genetic Variants. Preprint at 10.21203/rs.3.rs-668666/v1 (2021).

The original Article has been corrected.

