# Supplementary material for: Identification of loci associated with pathological outcomes in Holstein cattle infected with Mycobacterium avium subsp. paratuberculosis using whole-genome sequence data
Source: Sci Rep. 2021 Oct 11;11:20177. doi: 10.1038/s41598-021-99672-4 (PMC8505495; doi:10.1038/s41598-021-99672-4)
Supplement: Supplementary file 1 — Supplementary Information. [file 41598_2021_99672_MOESM1_ESM.pdf]

Identification of loci associated with pathological outcomes in Holstein cattle infected with *Mycobacterium avium* subsp. paratuberculosis using whole-genome sequence data

Supplementary Fig 1. Genomic distribution of the SNPs surpassing the threshold ($P < 5 \times 10^{-7}$) for evidence of an association with PTB-associated pathology. The chart depicts the genomic distribution of the SNPs associated with (A) multifocal and (B) diffuse lesions according to the Ensembl Variant Effect Predictor (VEP).

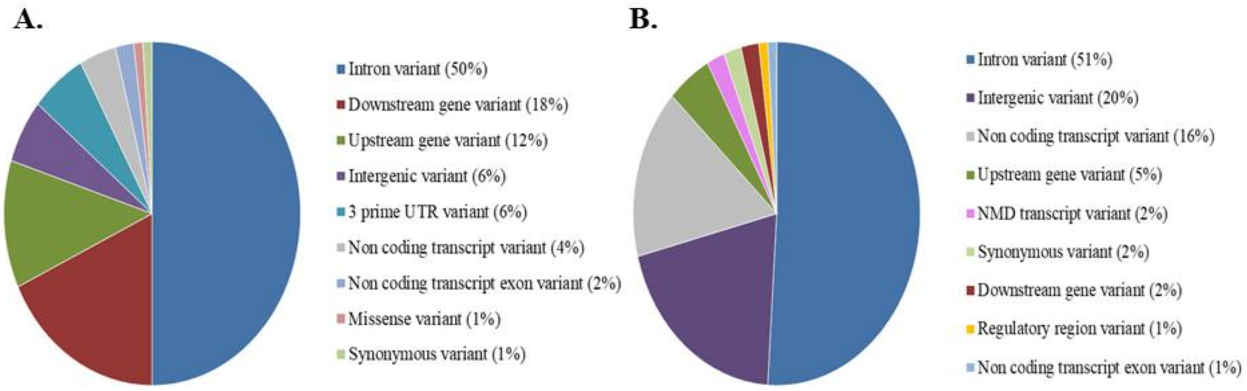

Supplementary Table 1. Overlaping of the multifocal lesions-associated QTLs identified in the current study with QTLs associated with other traits.

QTL	Bovine respiratory disease susceptibility	Somatic cell score	Tick resistance	Length of productive life	PTB susceptibility	Bovine Tuberculosis susceptibility	Clinical mastitis	IgG level
3:24233907-25325344								
5:24941743-26254968	QTL:160063	QTL:40818	QTL:9915					
5:38788039-39790008		QTL:175693, QTL:2659	QTL:9915					
5:23274617-24292059	QTL:160062	QTL:175680, QTL:10108	QTL:9915					
5:27260957-28354826		QTL:175681, QTL:2659	QTL:9915	QTL:123360	QTL:14844			
11:12914642-14422761		QTL:45161, QTL:1693				QTL:96711	QTL:4953	QTL:66186
11:5223771-6618872		QTL:1693	QTL:101151, QTL: 101166		QTL:169911, QTL: 169912, QTL:169913		QTL:5450	
11:9416584-10698629		QTL:1693		QTL:45103, QTL:45119			QTL:4953	QTL:66186
22:5865513-6865513								
22:2638700-4937859		QTL:178147, QTL:178282, QTL:178371, QTL:178467		QTL:51530				
22:1280377-2574848		QTL:178147, QTL:178282, QTL:178371, QTL:178467						
23:17326599-18326599								QTL:66220, QTL:66222, QTL:66217, QTL:66218, QTL:20493, QTL:66221, QTL:66215, QTL:66214, QTL:66219
24:37183179-38191028		QTL:122075						QTL:66223, QTL:66224, QTL:66225

Supplementary Table 2. Overlaping of the diffuse lesions-associated QTLs identified in the current study with QTLs associated with other traits.

QTLs	Bovine respiratory disease susceptibility	Clinical mastitis	Bovine tuberculosis susceptibility	Length of productive life	Somatic cell score	Tick resistance	Ig G level	PTB susceptibility
1:97006475-98111140	QTL:160000							
1:93955885-94925704					QTL:122054, QTL:122078			
3:63439078-64439078		QTL:2489	QTL:167791					
7:15613384-17275615	QTL:160105, QTL:160106		QTL:178961	QTL:42783	QTL:42787, QTL:3537	QTL:9916		
7:26041927-27059884					QTL:4683	QTL:9916	QTL:20475	
7:23796173-25086968	QTL:57631			QTL:42879		QTL:9916		QTL:166688
8:77894983-78394983								
13:66829928-68799183				QTL:31656				
23:13569222-14569289		QTL:164913	QTL:96552	QTL:106422, QTL:52010, QTL:52021, QTL:52025, QTL:52029, QTL:52031, QTL:52033, QTL:52035, QTL:52041, QTL:52050, QTL:52058,	QTL:52051, QTL:52059, QTL:52042		QTL:66220, QTL:66222, QTL:66217, QTL:66218, QTL:66221, QTL:66216, QTL:66215, QTL:66214, QTL:66219	

Supplementary Table 3. Frequency of cases and controls and odds ratios calculated under the codominant genetic model for the top 5 SNPs associated with PTB-associated lesions ($P < 5 \times 10^{-7}$).

Phenotype	BTA	SNP position	Genotype	Cases (%)	Controls (%)	OR (95% CI)
Multifocal	22	3,138,700	C/C	28 (84.8)	371 (99.5)	1.0
			T/C	5 (15.2)	2 (0.5)	33.48 (6.18-181.4)
	22	3,138,706	T/T	28 (84.8)	371 (99.5)	1.0
			G/T	5 (15.2)	2 (0.5)	33.48 (6.18-181.4)
	22	3,138,710	C/C	28 (84.8)	371 (99.5)	1.0
			T/C	5 (15.2)	2 (0.5)	33.48 (6.18-181.4)
	22	3,138,717	C/C	28 (84.8)	371 (99.5)	1.0
			T/C	5 (15.2)	2 (0.5)	33.48 (6.18-181.4)
	22	3,138,734	C/C	28 (84.8)	371 (99.5)	1.0
			T/C	5 (15.2)	2 (0.5)	33.48 (6.18-181.4)
Diffuse	13	67,404,014	A/A	18 (50.0)	323 (86.6)	1.0
			C/A	15 (41.7)	49 (13.1)	5.99 (2.79-12.89)
			C/C	3 (8.3)	1 (0.3)	50.08 (4.88-513.51)
	13	67,329,928	T/T	19 (52.8)	328 (87.9)	1.0
			C/T	14 (38.9)	44 (11.8	5.73 (2.64-12.41)
			C/C	3 (8.3)	1 (0.3)	47.75 (4.67-487.77)
	13	67,403,427	T/T	19 (52.8)	324 (86.9)	1.0
			G/T	14 (38.9)	48 (12.9)	5.26 (2.44-11.35)
			G/G	3 (8.3)	1 (0.3)	47.30 (4.63-483.30)
	8	78,394,983	C/C	31 (86.1)	371 (99.5)	1.0
			T/C	5 (13.9)	2 (0.5)	34.29 (6.03-195.1)
	8	78,394,984	T/T	31 (86.1)	371 (99.5)	1.0
			G/T	5 (13.9)	2 (0.5)	34.29 (6.03-195.1)

95 % CI- 95 % confidence interval
